# Short-and long-term outcomes of laparoscopic versus open gastrectomy in patients with gastric cancer: a systematic review and meta-analysis of randomized controlled trials

**DOI:** 10.1186/s12957-022-02818-5

**Published:** 2022-12-24

**Authors:** Xiaokang Lei, Yinkui Wang, Fei Shan, Shuangxi Li, Yongning Jia, Rulin Miao, Kan Xue, Zhemin Li, Jiafu Ji, Ziyu Li

**Affiliations:** Key Laboratory of Carcinogenesis and Translational Research (Ministry of Education/Beijing), Gastrointestinal Cancer Center, Peking University Cancer Hospital and Institute, 52 Fucheng Road, Haidian District, Beijing, 100142 China

**Keywords:** Short-term outcomes, Long-term outcomes, Laparoscopic gastrectomy, Open gastrectomy, Meta-analysis, Randomized controlled trials

## Abstract

**Background:**

Laparoscopic gastrectomy (LG) for gastric cancer has rapidly developed and become more popular in recent decades. Additional high-quality randomized controlled trial (RCT) studies comparing LG versus open gastrectomy (OG) for gastric cancer (GC) have been published in recent years. An updated systematic review is warranted. The aim of our meta-analysis was to comprehensively evaluate the short- and long-term outcomes of LG versus OG for GC.

**Materials and methods:**

The PubMed, Embase, Web of Science, and Cochrane Center Register of Controlled Trials databases were comprehensively searched to identify RCTs comparing LG versus OG for GC published between January 1994 and December 7, 2021. This study was conducted in accordance with the Preferred Reporting Items for Systematic Reviews and Meta-Analyses (PRISMA) and Cochrane Collaboration and the Quality of Reporting of Meta-analyses (QUORUM) guidelines. All RCTs comparing the short- and long-term outcomes of LG with those of OG were included. A random effects model was adopted with significant heterogeneity (*I*^2^ > 50%), while a fixed effects model was employed in all other cases (*I*^2^ ≤ 50%).

**Results:**

A total of 26 RCTs with 8301 patients were included in this meta-analysis. The results indicated that the intraoperative complication rate was comparable between the LG group and the OG group (OR=1.14, 95% CI [0.76, 1.70], *I*^2^=0%, *p*=0.53). The LG group had fewer postoperative complications than the OG group (OR=0.65, 95% CI [0.57, 0.74], *I*^2^=26%, *p*<0.00001). However, the severe postoperative complication rate and perioperative mortality were comparable between the two groups (OR=0.83, 95% CI [0.67, 1.04], *I*^2^=10%, *p*=0.10; OR=1.11, 95% CI [0.59, 2.09], *I*^2^=0%, *p*=0.74, respectively). The number of lymph nodes retrieved by the LG group was less than that of the OG group (MD=−1.51, 95% CI [−2.29, −0.74], *I*^2^=0%, *p*<0.0001). The proximal resection margin distance in the LG group was shorter than that in the OG group (MD=−0.34, 95% CI [−0.57, −0.12], *I*^2^=23%, *p*=0.003), but the distal resection margin distance in the two groups was comparable (MD=−0.21, 95% CI [−0.47, 0.04], *I*^2^=0%, *p*=0.10). The time to first ambulation was shorter in the LG group than in the OG group (MD=−0.14, 95% CI [−.26, −0.01], *I*^2^=40%, *p*=0.03). The time to first flatus was also shorter in the LG group than in the OG group (MD=−0.15, 95% CI [−0.23, −0.07], *I*^2^=4%, *p*=0.0001). However, the first time on a liquid diet was comparable between the two groups (MD=−0.30, 95% CI [−0.64, 0.04], *I*^2^=88%, *p*=0.09). Furthermore, the postoperative length of stay was shorter in the LG group than in the OG group (MD=−1.26, 95% CI [−1.99, −0.53], *I*^2^=90%, *p*=0.0007). The 5-year overall survival (OS) was comparable between the two groups (HR=0.97, 95% CI [0.80, 1.17], *I*^2^=0%, *p*=0.73), and the 5-year disease-free survival (DFS) was also similar between the LG group and OG group (HR=1.08, 95% CI [0.77, 1.52], *I*^2^=0%, *p*=0.64).

**Conclusion:**

LG is a technically safe and feasible alternative to OG with the advantages of a fewer postoperative complication rate, faster recovery of gastrointestinal function, and greater cosmetic benefit for patients with GC. Meanwhile, LG has comparable long-term outcomes to OG for GC.

**Supplementary Information:**

The online version contains supplementary material available at 10.1186/s12957-022-02818-5.

## Introduction

Gastric cancer (GC) is one of the most common digestive malignant tumors worldwide, and it had the fifth-highest morbidity rate and the fourth-highest mortality rate in 2020 [1]. Surgery is still the most effective treatment for GC [2]. Since Kitano et al. reported the first laparoscopy-assisted distal gastrectomy for early gastric cancer (EGC) in 1994 [3], this procedure has gained popularity globally [4–6]. Although several relevant meta-analyses have been conducted regarding short- and long-term outcomes of LG versus OG so far [7–11], most of these meta-analyses only included a few small RCTs or included non-RCTs. All these certain limitations lead to undermine the credibility of definitive conclusions. Besides, some important RCTs comparing LG to OG for GC have been published in the past several years, such as the CLASS-02 study, the largest-scale multicenter RCT focused on LTG versus OTG in China [12]; the STOMACH study, the largest-scale multicenter RCT regarding LTG compared to OTG after neoadjuvant chemotherapy (NACT) in European countries [13]; and the first RCT of LDG versus ODG after NACT in China [14]. Thus, it is necessary to conduct an updated and comprehensive systematic review and meta-analysis to compare the short- and long-term outcomes of LG versus OG among patients with GC. In this systematic review and meta-analysis, we just included high-quality RCTs, which made our conclusions more credible. Additionally, this meta-analysis contained more comprehensive outcome measures, such as intraoperative complications, postoperative complications, postoperative recovery, and long-term survival outcomes, making the study more complete. Moreover, we also focused on reducing the bias and identifying sources of significant heterogeneity to make the results more stable and reliable.

## Materials and methods

This systematic review and meta-analysis was conducted in accordance with the Preferred Reporting Items for PRISMA guidelines [15]. The protocol was registered on the PROSPERO website with the registration number CRD42022296300. The Participants, Intervention, Comparison, Outcome, Study design (PICOS) criteria are provided in Additional file 1*.*

### Search strategy

Two authors independently performed a comprehensive search of the PubMed, Web of Science, Embase, and Cochrane Center Register of Controlled Trials databases to identify relevant RCTs published in English between January 1994 and December 7th, 2021 (search date: December 7th, 2021). The keywords used were “gastric cancer,” “laparoscopic gastrectomy,” and “open gastrectomy.” Then, we combined these keywords and their mesh terms to form the search strategies. The search strategies are listed in Additional file 2. Moreover, the reference lists of included articles and previously published reviews were also screened. The search results were imported into EndNote (EndNote version X9 Clarivate Analytics). If there were two or more references regarding the short-term and long-term outcomes from the same authors or institutions, all references were included, and these references were regarded as the same study.

### Inclusion and exclusion criteria

Studies were screened in accordance with inclusion and exclusion criteria. The inclusion criteria were as follows: (1) patients diagnosed with GC by gastroscopic biopsy pathology or endoscopy; (2) patients who underwent laparoscopic gastrectomy, laparoscopic-assisted gastrectomy, or open gastrectomy; and (3) RCTs.

The exclusion criteria were as follows: (1) patients were diagnosed with a digestive system tumor other than GC, including gastrointestinal stromal tumors, neuroendocrine tumors, or benign lesions; (2) conference abstracts; (3) studies of hand-assisted laparoscopic gastrectomy or robotic gastrectomy; and (4) studies that did not provide adequate data.

### Literature screening and study selection

After identifying potentially relevant RCTs, we first removed duplicates using Endnote’s duplication filters. Subsequently, two researchers independently reviewed the titles and abstracts. Then, two authors (Xiaokang Lei and Yinkui Wang) independently read the full texts of the potentially relevant articles. Disagreements between the reviewers were resolved by consulting a third author (Fei Shan).

### Outcome definition

The intraoperative complications, overall postoperative complications, the rate of each type of postoperative complication, severe postoperative complications, and perioperative mortality were collected. Severe postoperative complications were defined as those with a Clavien–Dindo grade of III or greater or those defined as severe in the original articles. The surgical indicators were also recorded for analysis, including operation time and estimated blood loss (EBL). In addition, the surgical radicalness indices were collected, including the number of retrieved lymph nodes (LNs), proximal resected margin, and distal resected margin. Postoperative recovery indices were also recorded, including time to the first ambulation, time to first flatus, first time on a liquid diet, and length of hospital stay. Furthermore, survival outcomes, including the 5-year OS and 5-year DFS rates, were collected and analyzed.

### Quality and risk of bias assessment

Two reviewers (Xiaokang Lei and Yinkui, Wang) independently assessed the quality of the included trials, and disagreements were resolved through discussion. We evaluated the risk of bias for each study according to the Cochrane Handbook for Systematic Reviews of Interventions [16]. It contains the risk of selection bias (random sequence generation and allocation concealment), performance bias (blinding of participants and personnel), detection bias (blinding of outcome assessment), attrition bias (incomplete outcome data), reporting bias (selective reporting), and other bias. Each item was judged as “Low risk,” “High risk,” or “Unclear risk.”

### Data extraction

An electronic case-report form was used to collect data from each article. The following data were extracted: (1) study characteristics, including title, authors, country, publishing time, study design, method of randomization, blinding method, the surgical approach of each arm, and sample size in each arm; (2) patient characteristics, including age, sex, BMI, ASA scores, long tumor diameter, short tumor diameter, and tumor location; (3) primary outcomes, including the number of overall postoperative complications in each arm, classifications of postoperative complications, and the number of postoperative complications in each classification; (4) secondary outcomes, including the number of severe postoperative complications, the number of perioperative deaths, operation time, EBL, the number of retrieved lymph nodes, proximal resected margin, distal resected margin, time to first ambulation, time to first flatus, the first time on a liquid diet, and length of hospital stay; and (5) survival outcomes, including hazard ratio (HR), 95% confidence interval (CI), *p* value of 5-year OS and 5-year DFS, and survival curves. These data were extracted into Excel spreadsheets (Microsoft Corp., Redmond, WA, USA). The data were checked by two authors (Xiaokang Lei and Yinkui Wang).

### Statistical analysis

We conducted this meta-analysis following the QUORUM guidelines [17, 18]. Review Manager Version 5.3 (The Cochrane Collaboration, Software Update, Oxford, UK) was used for statistical analyses. The mean differences (MDs) with 95% CIs were used to analyze data for continuous variables. For dichotomous data, we calculated odds ratios (ORs) with 95% CIs. Hazard ratios (HRs) with 95% CIs were calculated for survival data. For survival data reported as survival curves without HRs, HRs were calculated with 95% CIs by the software Engauge Digitizer 11.1 through the methods described by Tierney in 2007 [19]. Heterogeneity was measured by *I*^2^ and Q statistics, and an *I*^2^ value greater than 50% indicated considerable heterogeneity. A random effects model was adopted when significant heterogeneity was observed (*I*^2^ > 50%), while a fixed effects model was employed in all other cases (*I*^2^ ≤ 50%). Sensitivity analysis was performed to identify sources of significant heterogeneity by removing each study from the meta-analysis one at a time. A funnel plot was used to evaluate the publication bias. Subgroup analysis was conducted based on country, type of gastrectomy, and tumor stage. Countries consisted of China, Japan, Republic of Korea (ROK), and Western countries. Types of gastrectomy included distal gastrectomy, total gastrectomy, and undifferentiated, which indicated that there was an undifferentiated type of gastrectomy in the original articles. Tumor stages included EGC or stage I gastric cancer, AGC, and mixture, which represented EGC or stage I gastric cancer plus AGC.

## Results

**Fig. 1:**
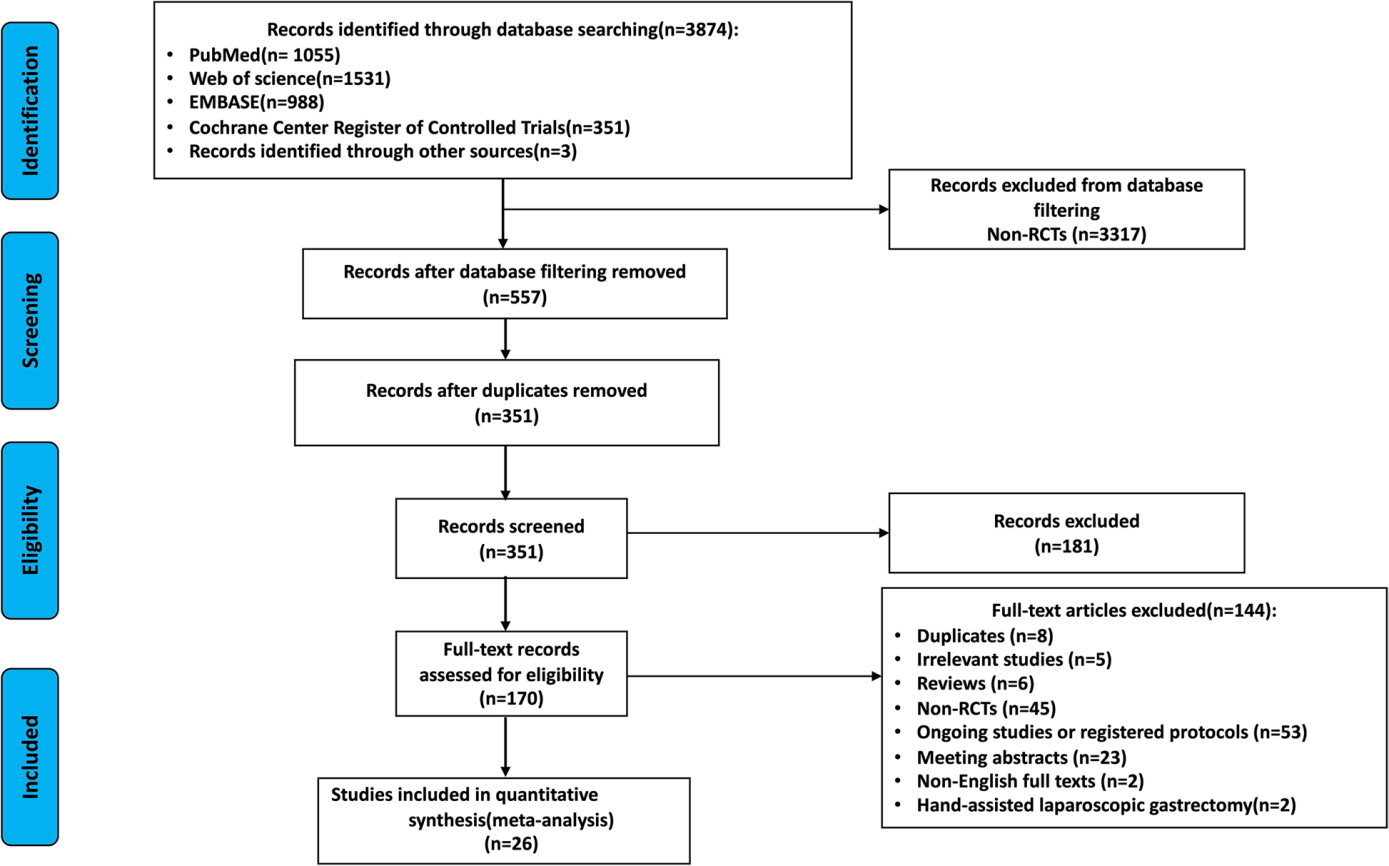
Study flow diagram showing selection of articles for analysis

After the comprehensive search, 3874 articles were initially identified. A total of 351 publications remained after removing duplicates and excluding the non-RCTs through the filtering function of the databases. Among these remaining articles, 181 publications were excluded after reviewing the title and abstract. Then, 170 articles remained for further assessment through full-text review, and 144 studies were excluded. Finally, 26 studies were included in the meta-analysis. The literature selection process is presented as the PRISMA flow diagram in Fig. 1.

### Characteristics of included studies

]The 26 enrolled studies involved 8301 patients, of whom 4211 underwent LG and 4090 underwent OG. These studies were published from 2002 to 2021 and included 15 single-center studies [5, 12, 14, 20–33] and 11 multicenter RCTs [6, 13, 34–47]. The detailed characteristics of the included studies are provided in Table 1.

**Table 1:** Characteristics of the included studies

Number	Studies	Country	Centers	Study period		Patients		Gender			
				Start	End	LG	OG	LG		OG	
								Male	Female	Male	Female
1	Cai et al. (2011) [21]	China	Single-center	2008.3	2009.12	49	47	39	10	37	10
2	Chen et al. (2012) [46]	China	Multi-centers	2009.1	2011.5	41	41	20	21	21	20
3	Cui et al. (2015) [20]	China	Single-center	2010.1	2012.12	128	142	88	40	98	44
4	Hayashi et al. (2005) [32]	Japan	Single-center	1999.12	2001.11	14	14	9	5	13	1
5^a^	Hu et al. (2016) [45]	China	Multi-centers	2012.9	2014.12	519	520	380	139	346	174
5^a^	Huang et al. (2022) [34]	China	Multi-centers	2012.9	2014.12	519	520	380	139	346	174
5^a^	Yu et al. (2019) [38]	China	Multi-centers	2012.9	2014.12	519	520	380	139	346	174
6	Huscher et al. (2005) [5]	Italy	Single-center	1992.11	1996.2	30	29	18	12	21	8
7	Hyung et al. (2020) [37]	Republic of Korea	Multi-centers	2011.11	2015.4	492	482	351	141	335	147
8^a^	Katai et al. (2017) [43]	Japan	Multi-centers	2010.3.15	2013.11.29	457	455	289	173	275	184
8^a^	Katai et al. (2020) [36]	Japan	Multi-centers	2010.3.15	2013.11.29	457	455	289	173	275	184
9^a^	Kim et al. (2008) [30]	Republic of Korea	Single-center	2003.7	2005.11	82	82	47	35	52	30
9^a^	Kim et al. (2013) [29]	Republic of Korea	Single-center	2003.7	2005.11	82	82	47	35	52	30
10	Kim et al. (2010) [47]	Republic of Korea	Multi-centers	2006.1.1	207.7.19	179	61	116	63	111	52
11^a^	Kim et al. (2016) [44]	Republic of Korea	Multi-centers	2006.2.1	2010.8.31	644	612	425	219	412	200
11^a^	Kim et al. (2019) [41]	Republic of Korea	Multi-centers	2006.1.5	2010.8.23	673	686	448	225	458	228
12	Kitano et al. (2002) [33]	Japan	Single-center	1998.10	2001.3	14	14	9	5	8	6
13	Lee et al. (2005) [31]	Republic of Korea	Single-center	2001.11	2003.8	24	23	11	13	15	8
14	Lee et al. (2019) [40]	Republic of Korea	Multi-centers	2011.11	2015.4	513	498	370	143	346	152
15	Li et al. (2019) [14]	China	Single-center	2015.4.23	2017.11.16	47	48	33	14	33	15
16	Liu et al. (2020) [12]	China	Single-center	2017.1	2018.10	105	109	75	30	80	29
17	Park et al. (2018) [42]	Republic of Korea	Multi-centers	2010.6	2011.10	100	96	69	31	65	31
18	Sakuramoto et al. (2013) [28]	Japan	Single-center	2005.10	2008.2	31	32	14	17	7	25
19	Sayed et al. (2021) [22]	Egypt	Single-center	2017.1	2019.12	16	20	13	7	11	5
20^a^	Shi et al. (2018) [24]	China	Single-center	2010.1	2012.6	162	160	120	42	105	55
20^a^	Shi et al. (2019) [23]	China	Single-center	2010.1	2012.6	161	156	120	41	101	55
21	Takiguchi et al. (2013) [27]	Japan	Single-center	2003.7	2006.1	20	20	12	8	13	7
22	van der Veen et al. (2021) [35]	Netherlands	Multi-centers	2015	2018	115	110	68	47	72	38
23	van der Wielen et al. (2021) [13]	Netherlands	Multi-centers	2015.1	2018.6	47	49	28	19	32	17
24	Wang et al. (2019) [39]	China	Multi-centers	2014.3	2017.8	222	220	144	78	133	87
25	Yamashita et al. (2016) [26]	Japan	Single-center	2005.1	2008.2	31	32	14	17	7	25
26	Zhou et al. (2017) [25]	China	Single-center	2012	2015	100	100	50	50	50	50

^a^These articles reported the results of the same study from the same authors or institutions

### Intraoperative complications, postoperative complications, and perioperative mortality

**Fig. 2:**
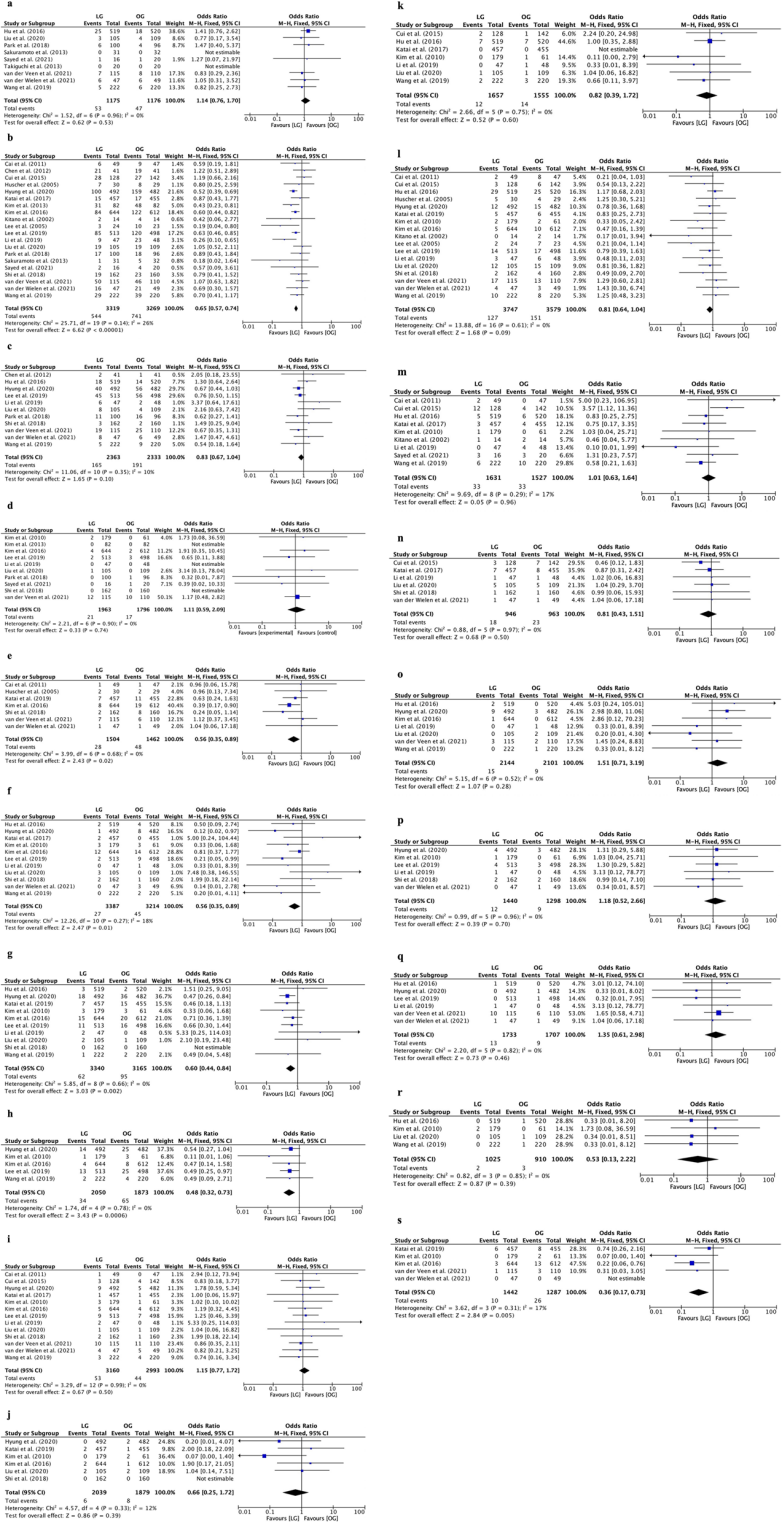
Forest plot between laparoscopic gastrectomy (LG) and open gastrectomy (G) group on intraoperative complications, postoperative complications, and perioperative mortality. **a** Intraoperative complication. **b** Overall postoperative complications. **c** Severe postoperative complications. **d** Perioperative mortality. **e** Surgical site complications. **f** Intra-abdominal bleeding. **g** Intestinal obstruction and ileus. **h** Intra-abdominal fluid collection. **i** Anastomotic leakage. **j** Anastomotic stenosis. **k** Chyle leak. **l** Pulmonary complications. **m** Delayed gastric emptying. **n** Abdominal infections/abscess. **o** Pancreatic leakage. **p** Urinary tract infection. **q** Cardiac complications. **r** Renal complications. **s** Wound dehiscence/hernia

Among all the enrolled articles, seven studies reported intraoperative complications. The analysis showed that the intraoperative complication rate was comparable between the LG and OG groups (OR=1.14, 95% CI [0.76, 1.70], *I*^2^=0%, *p*=0.53; Fig. 2a). The overall postoperative complications in the LG group were less than those in the OG group (OR=0.65, 95% CI [0.57, 0.74], *I*^2^=26%, *p*<0.00001; Fig. 2b). There were no differences in the rate of severe postoperative complications between the OG and LG groups (OR=0.83, 95% CI [0.67, 1.04], *I*^2^=10%, *p*=0.10; Fig. 2c). The perioperative mortality was also similar between the two groups (OR=1.11, 95% CI [0.59, 2.09], *I*^2^=0%, *p*=0.74; Fig. 2d). Compared to the OG group, surgical site complications, such as wound infection and wound dehiscence, were less common in the LG group (OR=0.56, 95% CI [0.35, 0.89], *I*^2^=0%, *p*=0.02; Fig. 2e). Moreover, the LG group had fewer cases of intra-abdominal bleeding than the OG group (OR=0.56, 95% CI [0.35, 0.89], *I*^2^=18%, *p*=0.01; Fig. 2f). In addition, the LG group showed lower rates of intestinal obstruction and ileus than the OG group (OR=0.60, 95% CI [0.44, 0.84], *I*^2^=0%, *p*=0.002; Fig. 2g). The LG group also experienced less intra-abdominal fluid collection than the OG group (OR=0.48, 95% CI [0.32, 0.73], *I*^2^=0%, *p*=0.0006; Fig. 2h). Other complications, including anastomotic leakage, anastomotic stenosis, chyle leakage, pulmonary complications, delayed gastric emptying, abdominal infections/abscesses, pancreatic leakage, urinary tract infection, cardiac complications, and renal complications, were not significantly different between the two groups (*p*>0.05). The detailed results are provided in Fig. 2*.*

### Surgical indicators

**Fig. 3:**
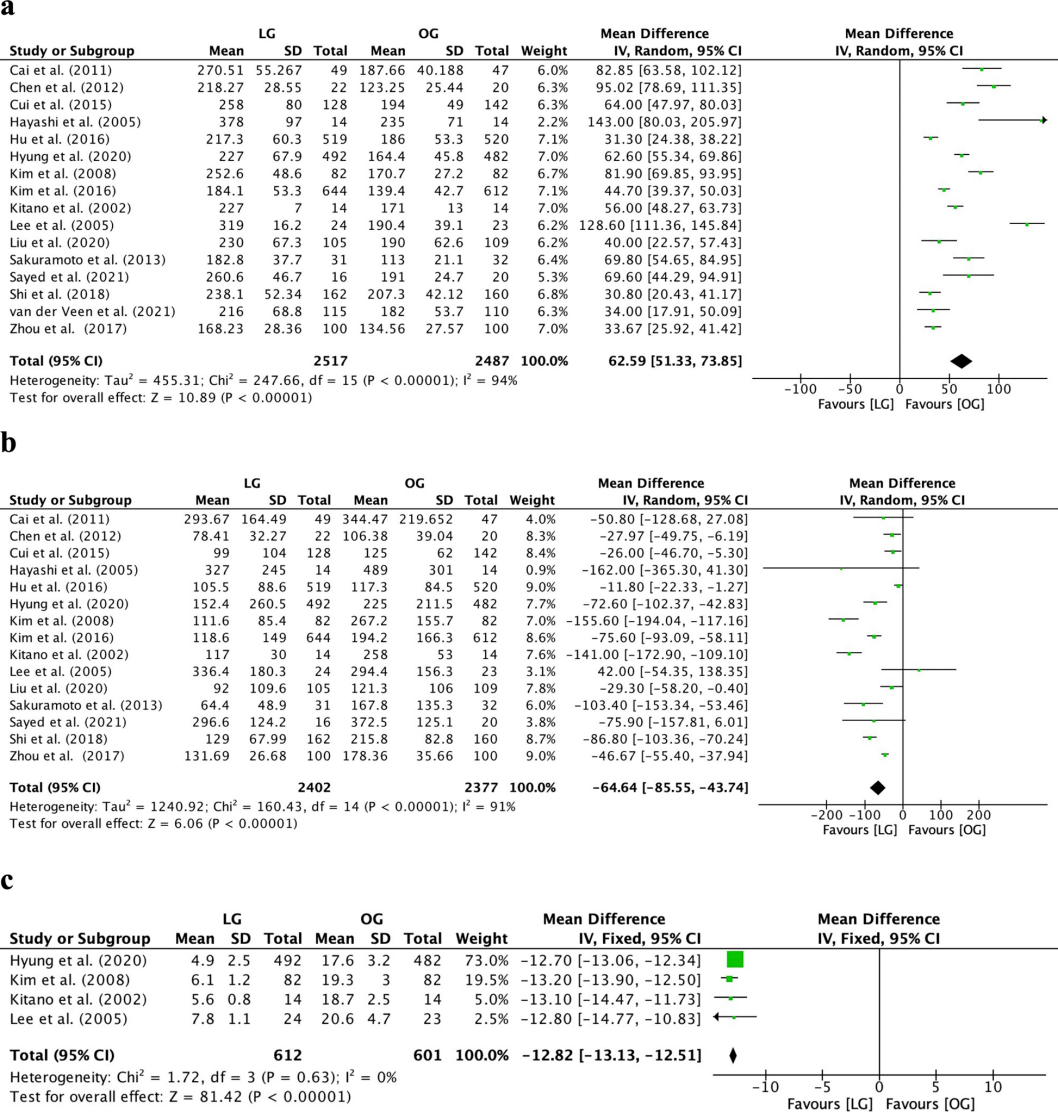
Forest plot between laparoscopic gastrectomy (LG) and open gastrectomy (OG) group on surgical indicators. **a** Operative time. **b** Estimated blood loss. **c** Length of incision

The operation time was significantly longer in the LG group than in the OG group (MD=62.59, 95% CI [51.33, 73.85], *I*^2^=94%, *p*<0.00001; Fig. 3a). However, EBL was significantly lower in the LG group than in the OG group (MD=−64.64, 95% CI [−85.55, −43.74], *I*^2^=91%, *p*<0.00001; Fig. 3b). Additionally, the LG group had significantly shorter incisions than the OG group (MD=−12.82, 95% CI [−13.13, −12.51], *I*^2^=0%, *p*<0.00001; Fig. 3c). These results are shown in Fig. 3.

### Surgical radicalness

**Fig. 4:**
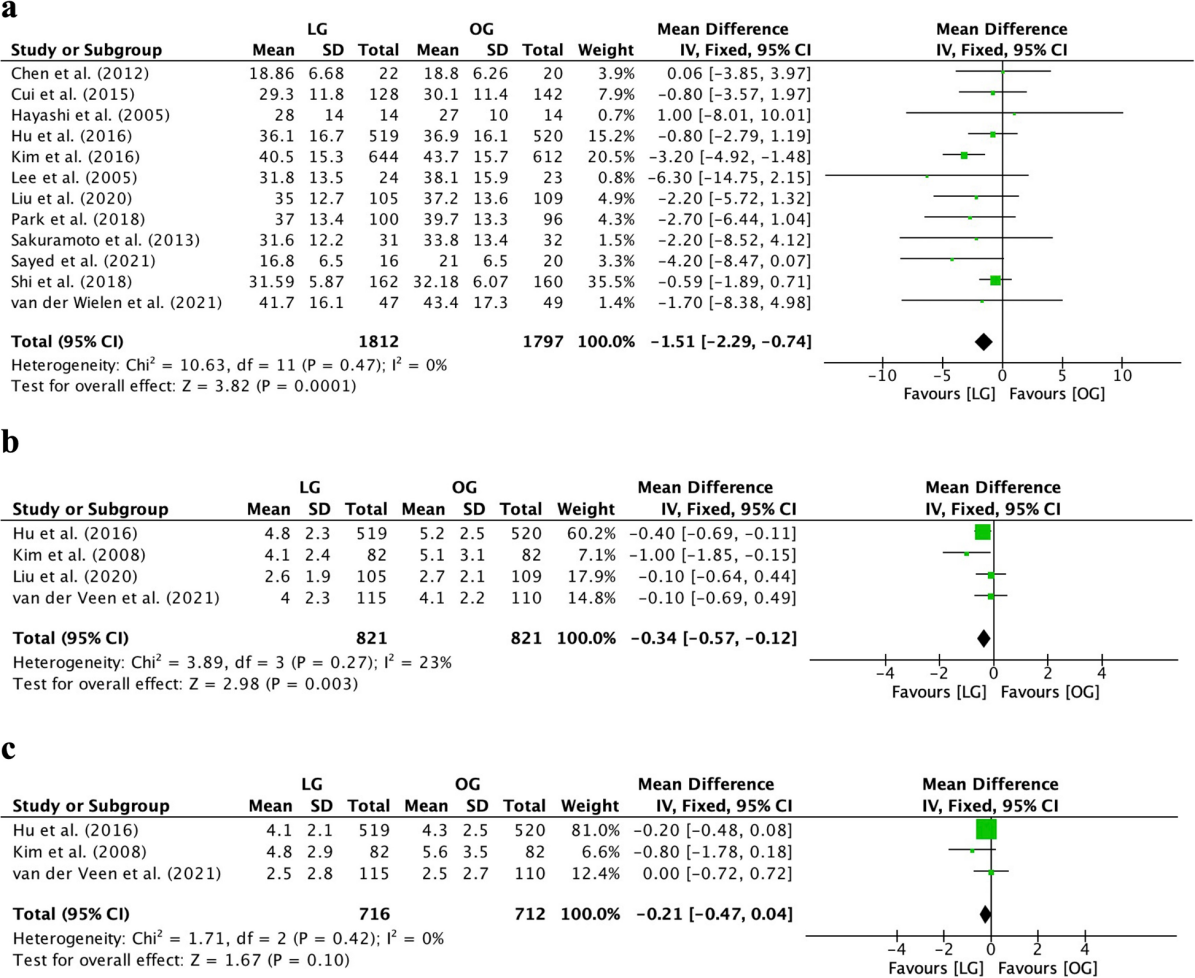
Forest plot between laparoscopic gastrectomy (LG) and open gastrectomy (OG) group on surgical radicalness. **a** The number of lymph nodes retrieved. **b** The proximal resection margin distance. **c** The distal resection margin distance

The number of lymph nodes retrieved from the LG group was less than that from the OG group (MD=−1.51, 95% CI [−2.29, −0.74], *I*^2^=0%, *p*=0.0001; Fig. 4a). The proximal resection margin distance in the LG group was shorter than that in the OG group (MD=−0.34, 95% CI [−0.57, −0.12], *I*^2^=23%, *p*=0.003, Fig. 4b), but the distal resection margin distance in the two groups was comparable (MD=−0.21, 95% CI [−0.47, 0.04], *I*^2^=0%, *p*=0.10, Fig. 4c). The detailed results are provided in Fig. 4.

### Postoperative recovery

**Fig. 5:**
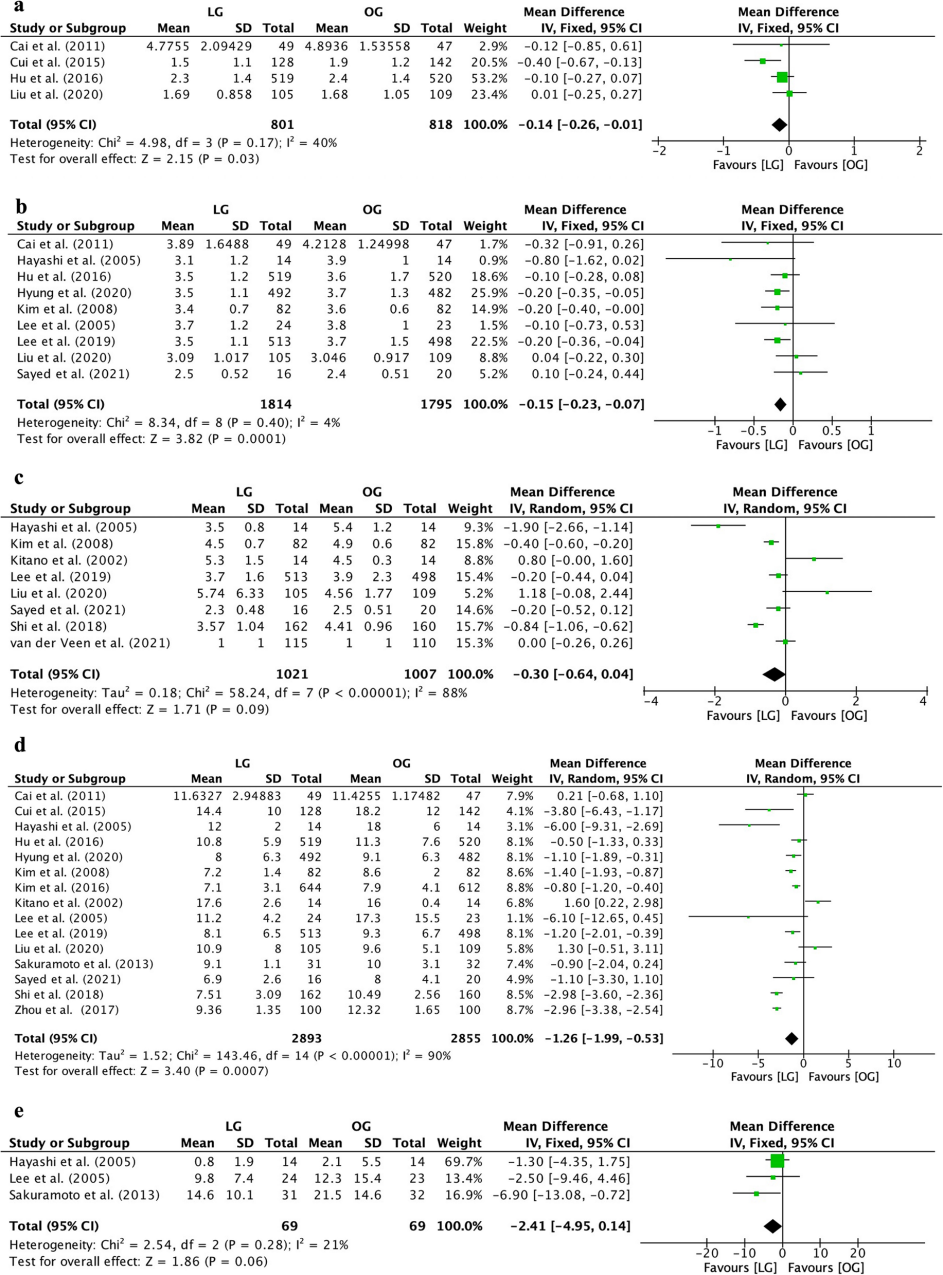
Forest plot between laparoscopic gastrectomy (LG) and open gastrectomy (OG) group on postoperative recovery. **a** The time to first ambulation. **b** The time to first flatus. **c** The first time on a liquid diet. **d** The postoperative length of stay. **e** The postoperative analgesic

The time to first ambulation was shorter in the LG group than in the OG group (MD=−0.14, 95% CI [−0.26, −0.01], *I*^2^=40%, *p*=0.03, Fig. 5a). The time to first flatus was also shorter in the LG group than in the OG group (MD=−0.15, 95% CI [−0.23, −0.07], *I*^2^=4%, *p*=0.0001, Fig. 5b). However, the first time on a liquid diet was comparable between the two groups (MD=−0.30, 95% CI [−0.64, 0.04], *I*^2^=88%, *p*=0.09, Fig. 5c). Furthermore, the postoperative length of stay was shorter in the LG group than in the OG group (MD=−1.26, 95% CI [−1.99, −0.53], *I*^2^=90%, *p*=0.0007, Fig. 5d). The number of postoperative analgesics used in the LG group was similar to that in the OG group (MD=−2.41, 95% CI [−4.95, 0.14], *I*^2^=21%, *p*=0.06, Fig. 5e).

### Survival outcomes

**Fig. 6:**
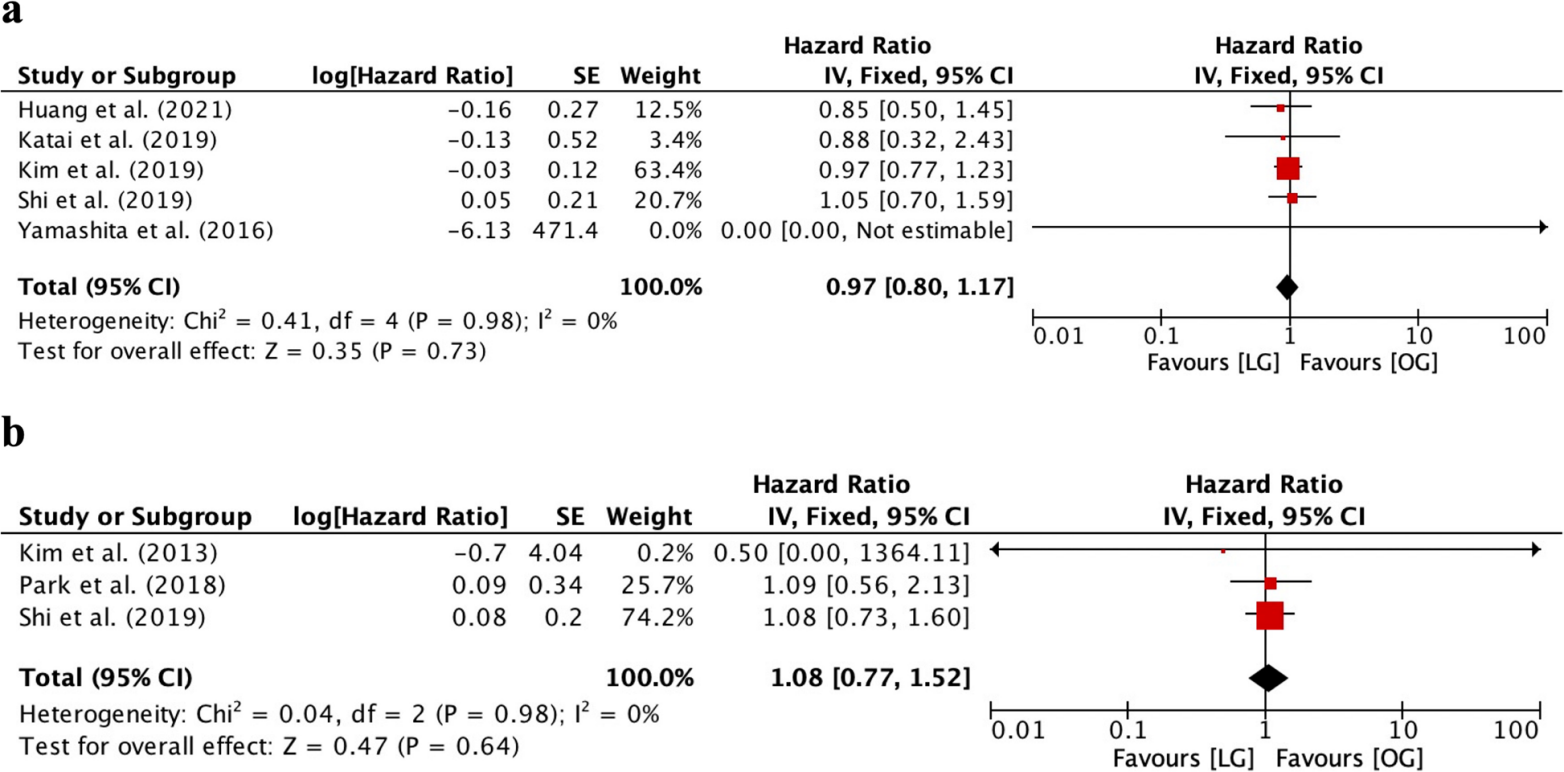
Forest plot between laparoscopic gastrectomy (LG) and open gastrectomy (OG) group on survival outcomes. **a** 5-year OS. **b** 5-year DFS

The study showed that the 5-year OS was similar between the two groups (HR=0.97, 95% CI [0.80, 1.17], *I*^2^=0%, *p*=0.73, Fig. 6a). The 5-year DFS was also comparable between the two groups (HR=1.08, 95% CI [0.77, 1.52], *I*^2^=0%, *p*=0.64, Fig. 6b).

### Quality and risk of bias assessment

**Fig. 7:**
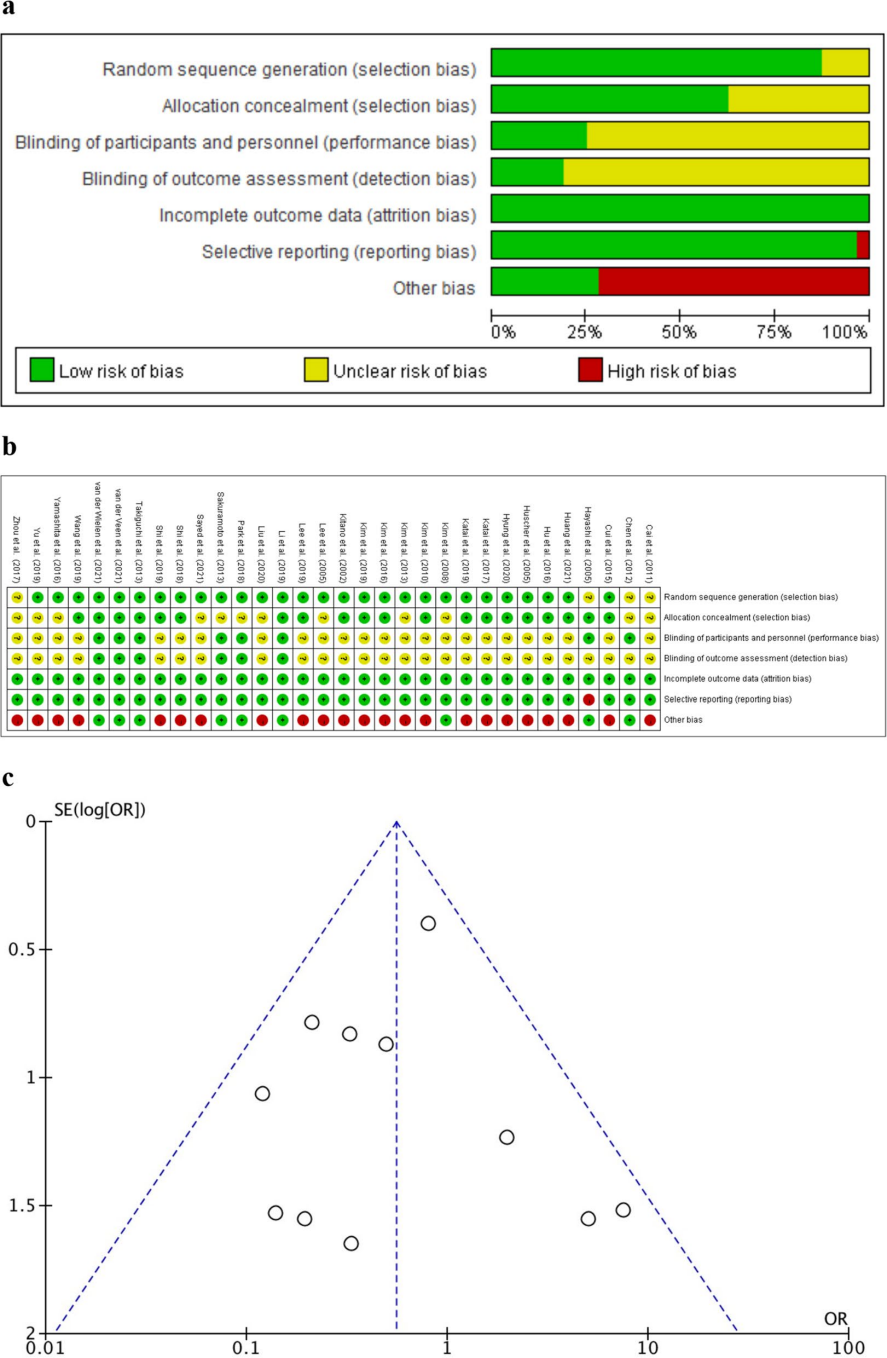
Risk of bias. **a** Risk of bias graph. **b** Risk of bias summary. **c** Funnel plot of sensitivity analysis

The results of the risk of bias, risk of bias summary, and risk of graph of the Cochrane Handbook for Systematic Reviews of Interventions are provided in Fig. 7. Publication bias was evaluated using a funnel plot, and the results are shown in Fig. 7*.*

### Subgroup analysis

Subgroup analysis showed that the operation time was also significantly longer in the LG group than in the OG group for each group. In addition, subgroup analysis showed that EBL was also significantly lower in the LG group than in the OG group in all subgroups. For the first time on a liquid diet, subgroup analysis suggested that the first time on a liquid diet was shorter in the LG group than in the OG group in the ROK (MD=−0.32, 95% CI [−0.47, −0.17], *I*^2^=35%, *p*<0.0001, Table 4). However, no differences in the first time on a liquid diet were observed between the LG group and the OG group in other subgroups. For postoperative length of stay, subgroup analysis showed that postoperative length of stay was shorter in the LG group than in the OG group in China and the ROK (China: MD=−1.42, 95% CI [−2.79, −0.06], *I*^2^=94%, *p*=0.04; ROK: MD=−1.06, 95% CI [−1.34, −0.78], *I*^2^=94%, *p*=0.04, Table 5). Similarly, subgroup analysis showed that postoperative length of stay was shorter in the LG group than in the OG group in subgroups of distal gastrectomy, no mention, EGC or stage I, and AGC (*p*<0.05, Table 5). The postoperative length of stay was comparable between the two groups in other subgroups (Table 5). Detailed results are provided in Tables 2, 3, 4, and 5.

**Table 2:** Subgroup analysis of operation time

Variables	Number of studies	Heterogeneity	Model	MD	95% CI		*P*	*P*_sub_
		*I*^2^			Lower	Upper		
Countries								0.30
China	7	93%	RE	52.76	36.52	69.01	<0.00001	
Japan	3	79%	RE	70.66	48.09	93.24	<0.00001	
ROK	4	97%	RE	78.29	52.02	104.57	<0.00001	
Type of gastrectomy								0.03
Distal gastrectomy	8	96%	RE	75.41	56.40	94.43	<0.00001	
Total gastrectomy	1	-	RE	40.00	22.57	57.43	<0.00001	
Undifferentiated^a^	7	90%	RE	52.63	38.00	67.26	<0.00001	
Stages								0.23
EGC or stage I	8	95%	RE	68.19	50.65	85.73	<0.00001	
AGC	4	94%	RE	47.06	27.43	66.68	<0.00001	
Mixture^b^	4	90%	RE	68.81	42.08	95.54	<0.00001	

*ROK* Republic of Korea, *EGC* early gastric cancer, *AGC* advanced gastric cancer, *P*_*sub*_*P*-value of subgroup

^a^Undifferentiated: there was undifferentiated of type of gastrectomy in original articles

^b^Mixture: EGC or stage I gastric cancer plus AGC

**Table 3:** Subgroup analysis of EBL

Variables	Number of studies	Heterogeneity	Model	MD	95% CI		*P*	*P*_sub_
		*I*^2^			Lower	Upper		
Countries								0.0007
China	7	91%	RE	−39.05	−60.33	−17.77	0.0003	
Japan	3	0%	FE	−130.65	−157.30	−104.00	<0.00001	
ROK	4	86%	RE	−80.13	−126.45	−33.80	0.0007	
Western countries	1	-	RE	−75.90	−157.81	6.01	0.07	
Type of gastrectomy								0.15
Distal gastrectomy	8	95%	RE	−75.38	−117.76	−33.00	0.0005	
Total gastrectomy	1	-	RE	−29.30	−58.20	−0.40	0.05	
^a^Undifferentiated	6	81%	RE	−58.10	−80.60	−35.61	<0.00001	
Stages								0.010
EGC or stage I	8	90%	RE	−81.76	−115.00	−48.52	<0.00001	
AGC	4	95%	RE	−59.62	−109.49	−9.74	0.02	
^b^Mixture	3	0%	FE	−27.79	−42.53	−13.05	0.0002	

*ROK* Republic of Korea, *EGC* early gastric cancer, *AGC* advanced gastric cancer, *P*_*sub*_*P*-value of subgroup

^a^Undifferentiated: there was undifferentiated of type of gastrectomy in original articles

^b^Mixture: EGC or stage I gastric cancer plus AGC

**Table 4:** Subgroup analysis of the first time on a liquid diet

Variables	Number of studies	Heterogeneity	Model	MD	95% CI		*P*	*P*_sub_
		*I*^2^			Lower	Upper		
Countries								0.42
China	2	90%	RE	0.07	−1.90	2.04	0.94	
Japan	2	96%	RE	−0.55	−3.20	2.09	0.68	
ROK	2	35%	FE	−0.32	−0.47	−0.17	<0.0001	
Western countries	2	0%	FE	−0.08	−0.28	0.12	0.45	
Type of gastrectomy								0.07
Distal gastrectomy	4	88%	RE	−0.41	−0.95	0.13	0.14	
Total gastrectomy	1	-	RE	1.18	−0.08	2.44	0.07	
^a^Undifferentiated	3	92%	RE	−0.35	−0.90	0.20	0.21	
Stages								0.29
EGC or stage I	4	90%	RE	−0.16	−1.20	0.89	0.77	
AGC	3	89%	RE	−0.42	−0.87	0.03	0.07	
^b^Mixture	1	-	RE	0.00	−0.26	0.26	1.00	

*ROK* Republic of Korea, *EGC* early gastric cancer, *AGC* advanced gastric cancer, *P*_*sub*_*P*-value of subgroup

^a^Undifferentiated: there was undifferentiated of type of gastrectomy in original articles

^b^Mixture: EGC or stage I gastric cancer plus AGC

**Table 5:** Subgroup analysis of postoperative length of stay

Variables	Number of studies	Heterogeneity	Model	MD	95% CI		*P*	*P*_sub_
		*I*^2^			Lower	Upper		
Countries								0.97
China	6	94%	RE	−1.42	−2.79	−0.06	0.04	
Japan	3	90%	RE	−1.36	−4.39	1.68	0.38	
ROK	5	28%	FE	−1.06	−1.34	−0.78	<0.00001	
Western countries	1	-	RE	−1.10	−3.30	1.10	0.33	
Type of gastrectomy								0.01
Distal gastrectomy	8	76%	RE	−0.91	−1.59	−0.23	0.009	
Total gastrectomy	1	-	RE	1.30	−0.51	3.11	0.16	
^a^Undifferentiated	6	91%	RE	−1.88	−3.05	−0.71	0.002	
Stages								0.95
EGC or stage I	8	93%	RE	−1.19	−2.32	−0.07	0.04	
AGC	5	86%	RE	−1.42	−2.47	−0.37	0.008	
^b^Mixture	2	88%	RE	−1.60	−5.51	2.31	0.42	

*ROK* Republic of Korea, *EGC* early gastric cancer, *AGC* advanced gastric cancer, *P*_sub_*P*-value of subgroup

^a^Undifferentiated: there was undifferentiated of type of gastrectomy in original articles

^b^Mixture: EGC or stage I gastric cancer plus AGC

## Discussion

LG has been thriving for nearly two decades since the first LDG was reported in 1994 [3]. Several RCTs comparing LG with OG for GC have been published in the past several years. This meta-analysis included patients who had distal gastrectomy or total gastrectomy for early gastric cancer (EGC) and advanced gastric cancer (AGC) with or without NACT treatment. A total of 26 RCTs were identified and included in this analysis. The present study is an updated meta-analysis with a larger sample size to evaluate the short- and long-term outcomes between LTG and OTG in patients with GC. In addition, this meta-analysis included several latest and important RCTs [12–14]. Furthermore, several famous RCTs, including CLASS-01, KLASS-01, and KLASS-02, have reported long-term survival outcomes in the past several years [34, 37, 38, 41]. The current meta-analysis also included these studies. Long-term outcomes are crucial for patients with GC and play a key role in selecting surgical approaches [48]. This present meta-analysis showed that 5-year OS and 5-year DFS were comparable between the LG group and OG group, which indicated that LG had comparable long-term safety versus OG for patients with GC. This study is also the first systematic review and meta-analysis evaluating long-term outcomes of LG versus OG in patients with GC.

This systematic review and meta-analysis showed that LG had advantages in significantly reducing surgical site complications, intra-abdominal bleeding, and intestinal obstruction or ileus, with no significant differences in anastomotic stenosis, anastomotic leakage, abdominal infection or abscess, chyle leak, pulmonary complications, or pancreatic leakage between the two groups. The present study is the first systematic review and meta-analysis reporting the intraoperative complications of LG versus OG among patients with GC. A previous meta-analysis reported that the rate of intra-abdominal bleeding in the LG group was comparable with that in the OG group (*p*>0.05) [49]. This result of our study is different from the previous meta-analysis. We found that the rate of intra-abdominal bleeding in the LG group was lower than that in the OG group (*p*=0.01). Compared with previous studies [49, 50], the present meta-analysis included a larger sample size of RCTs and provided more solid evidence. In addition, we first found that LG could decrease the intra-abdominal fluid collection rate compared to OG in a meta-analysis. In comparison with OG, LG could provide a magnified view for operation, which made the blood vessel and tissue clearer so that unnecessary damage can be avoided. To sum up, the present meta-analysis indicated that LG had same or better safety than OG in terms of intraoperative complications and overall postoperative complications.

The present meta-analysis showed that LG led to benefits regarding cosmetic appearance and led to less blood loss than OG, but it required a longer operation time. Notably, the number of lymph nodes retrieved from the LG group was less than that from the OG group, and the proximal resection margin distance in the LG group was shorter than that in the OG group. The number of lymph nodes retrieved is correlated with the prognosis of patients. However, the present meta-analysis showed that there was no significant difference in long-term outcomes between the LG group and OG group. Additionally, the study revealed that LG had notable benefits in less analgesic use, shorter time to the first ambulation, shorter time to first flatus, and shorter postoperative length of stay compared to OG. However, there was no significant difference in the first time on a liquid diet between the two groups, which was different from the previous meta-analysis. The study further supported that LG had advantages in quicker postoperative recovery than OG.

Sensitivity analysis was conducted to test the stability of the results and identify sources of significant heterogeneity. In addition, fixed and random effects models were also used to test the stability of the results for each comparison. The results suggested that the results of the present meta-analysis were stable. However, there were significant heterogeneities in the following four analyses, including operation time (*I*^2^=94%), EBL (*I*^2^=91%), the first time on a liquid diet (*I*^2^=88%), and the postoperative length of stay (*I*^2^=90%). Therefore, random effects models were selected. Additionally, we performed subgroup analysis based on country, type of gastrectomy, and tumor stage. The subgroup analysis results of operation time and EBL were in accordance with the overall analysis. Notably, the heterogeneity of EBL was decreased in the Japanese subgroup. Interestingly, some subgroup analysis results of the first time on a liquid diet and the postoperative length of stay were different from the results of the overall analysis. Subgroup analysis showed that the first time on a liquid diet was significantly shorter in the LG group than in the OG group among ROK patients with low heterogeneity (*I*^2^=35%). In addition, subgroup analysis indicated that the postoperative length of stay in the LG group was comparable to that in the OG group in Japan and Western countries, which was also different from the overall analysis result. These results indicated that there was heterogeneity among different countries. This could be due to different tumor features, such as the stage or grade, among different countries. Additionally, the meta-analysis enrolled studies regarding EGC or stage I and AGC, and the results were similar among each subgroup. The LDG has been recommended as a treatment option for clinical stage I distal GC by the guidelines, which indicated that the LG for other stages GC was also worth considering to be recommended as a treatment option by the guidelines.

## Limitations

Although this meta-analysis was conducted based on RCTs, there were several limitations. First, some data in some of these RCTs were missing due to the lack of data in the original studies. Second, most of these RCTs had a high risk of bias or unclear risk of bias in blinding and other bias because of the loss of detailed description in the original articles. Third, although we conducted sensitivity analysis and subgroup analysis, several results were still highly heterogeneous. Fourth, for survival analysis, some data were extracted through survival curves. Although two authors independently calculated the survival data according to the software Engauge Digitizer 11.1 followed by the standard methods of Tierney reported in 2007 [19], there would still exist bias. Last, some important parameters for cancer patients, such as health-related quality of life, were not included for analysis in this study.

## Conclusions

LG is a technically safe and feasible alternative to OG with the advantages of a fewer postoperative complication rate, faster recovery of gastrointestinal function, and greater cosmetic benefit for patients with GC. Meanwhile, LG has comparable long-term outcomes to OG for GC.

## Supplementary Information

**Additional file 1.** PICOS criteria for inclusion and exclusion of studies.

**Additional file 2.** Search strategies.

## Data Availability

The datasets generated and/or analyzed during the current study are publicly available. If you need relevant data after the article is published, you can contact the corresponding author.

## Abbreviations

LG: Laparoscopic gastrectomy
OG: Open gastrectomy
RCTs: Randomized controlled trials
OS: Overall survival
DFS: Disease-free survival
GC: Gastric cancer
LAGC: Laparoscopy-assisted distal gastrectomy
EGC: Early gastric cancer
AGC: Advanced gastric cancer
NACT: Neoadjuvant chemotherapy
PRISMA: Preferred Reporting Items for Systematic Reviews and Meta-Analyses
PICOS: Participants, Intervention, Comparison, Outcome, Study design
EBL: Estimated blood loss
LNs: Lymph nodes
QUORUM: Cochrane Collaboration and the Quality of Reporting of Meta-analyses
MDs: Mean differences
ORs: Odds ratios
HR: Hazard ratio
ROK: Republic of Korea
