# Supplementary material for: Short-and long-term outcomes of laparoscopic versus open gastrectomy in patients with gastric cancer: a systematic review and meta-analysis of randomized controlled trials
Source: World J Surg Oncol. 2022 Dec 24;20:405. doi: 10.1186/s12957-022-02818-5 (PMC9789553; doi:10.1186/s12957-022-02818-5)
Supplement: Supplementary file 1 — Additional file 1. PICOS criteria for inclusion and exclusion of studies. [file 12957_2022_2818_MOESM1_ESM.docx]

Additional file 1. PICOS criteria for inclusion and exclusion of studies

| Parameters | Inclusion criteria | Exclusion criteria |
| --- | --- | --- |
| Patients | Patients underwent gastrectomy with/without D2 lymphadenectomy for gastric cancer |  |
| Intervention | Laparoscopic gastrectomy (LG)  Laparoscopic-assisted gastrectomy (LAG) | Robotic gastrectomy  Hand-assisted gastectomy |
| Comparator | Open gastrectomy |  |
| Outcomes | Primary outcome: overall postoperative complication rate, 5-year OS  Secondary outcomes: operation time, estimated blood loss, perioperative mortality, severe postoperative complication rate, number of retrieved lymph nodes, time to first flatus, time to first defecating, the first time on a liquid diet, postoperative mortality within 30 days after surgery, length of hospital stay, severe postoperative complication rate |  |
| Study design | RCT | Case report, Case series, Case control study, Prospective cohort study, Retrospective cohort study |
