# Supplementary material for: Short-and long-term outcomes of laparoscopic versus open gastrectomy in patients with gastric cancer: a systematic review and meta-analysis of randomized controlled trials
Source: World J Surg Oncol. 2022 Dec 24;20:405. doi: 10.1186/s12957-022-02818-5 (PMC9789553; doi:10.1186/s12957-022-02818-5)
Supplement: Supplementary file 2 — Additional file 2. Search strategies. [file 12957_2022_2818_MOESM2_ESM.docx]

Additional file 2 Search strategies

Additional file 2-1 Search strategy: PubMed

((((((((((((((((((((((Neoplasm, Stomach[Title/Abstract]) OR (Stomach Neoplasm[Title/Abstract])) OR (Neoplasms, Stomach[Title/Abstract])) OR (Gastric Neoplasms[Title/Abstract])) OR (Gastric Neoplasm[Title/Abstract])) OR (Neoplasm, Gastric[Title/Abstract])) OR (Neoplasms, Gastric[Title/Abstract])) OR (Cancer of Stomach[Title/Abstract])) OR (Stomach Cancers[Title/Abstract])) OR (Gastric Cancer[Title/Abstract])) OR (Cancer, Gastric[Title/Abstract])) OR (Cancers, Gastric[Title/Abstract])) OR (Gastric Cancers[Title/Abstract])) OR (Stomach Cancer[Title/Abstract])) OR (Cancer, Stomach[Title/Abstract])) OR (Cancers, Stomach[Title/Abstract])) OR (Cancer of the Stomach[Title/Abstract])) OR (Gastric Cancer, Familial Diffuse[Title/Abstract])) OR ("Stomach Neoplasms"[Mesh])) AND (((((((((((((((((((((Laparoscopies[Title/Abstract]) OR (Celioscopy[Title/Abstract])) OR (Celioscopies[Title/Abstract])) OR (Peritoneoscopy[Title/Abstract])) OR (Peritoneoscopies[Title/Abstract])) OR (Surgical Procedures, Laparoscopic[Title/Abstract])) OR (Laparoscopic Surgical Procedure[Title/Abstract])) OR (Procedure, Laparoscopic Surgical[Title/Abstract])) OR (Procedures, Laparoscopic Surgical[Title/Abstract])) OR (Surgery, Laparoscopic[Title/Abstract])) OR (Laparoscopic Surgical Procedures[Title/Abstract])) OR (Laparoscopic Surgery[Title/Abstract])) OR (Laparoscopic Surgeries[Title/Abstract])) OR (Surgeries, Laparoscopic[Title/Abstract])) OR (Laparoscopic Assisted Surgery[Title/Abstract])) OR (Laparoscopic Assisted Surgeries[Title/Abstract])) OR (Surgeries, Laparoscopic Assisted[Title/Abstract])) OR (Surgery, Laparoscopic Assisted[Title/Abstract])) OR (Surgical Procedure, Laparoscopic[Title/Abstract])) OR ("Laparoscopy"[Mesh])) OR (((((((((((((((((Surgical Procedure, Minimal[Title/Abstract]) OR (Surgical Procedures, Minimal[Title/Abstract])) OR (Surgical Procedures, Minimal Access[Title/Abstract])) OR (Surgical Procedures, Minimally Invasive[Title/Abstract])) OR (Procedures, Minimally Invasive Surgical[Title/Abstract])) OR (Minimal Surgical Procedure[Title/Abstract])) OR (Minimal Surgical Procedures[Title/Abstract])) OR (Minimally Invasive Surgery[Title/Abstract])) OR (Minimally Invasive Surgeries[Title/Abstract])) OR (Surgeries, Minimally Invasive[Title/Abstract])) OR (Surgery, Minimally Invasive[Title/Abstract])) OR (Procedure, Minimal Surgical[Title/Abstract])) OR (Procedures, Minimal Access Surgical[Title/Abstract])) OR (Procedures, Minimal Surgical[Title/Abstract])) OR (Minimally Invasive Surgical Procedure[Title/Abstract])) OR (Minimal Access Surgical Procedures[Title/Abstract])) OR ("Minimally Invasive Surgical Procedures"[Mesh])))) AND ((Gastrectomies[Title/Abstract]) OR ("Gastrectomy"[Mesh]))) AND ((((open gastrectomy[MeSH Terms]) OR (open surgery[Title/Abstract])) OR (open operation[Title/Abstract])) OR (open approach[Title/Abstract])))

Additional file 2-2 Search strategy: EMBASE

(('stomach cancer'/exp) OR ('cancer of the stomach':ab,ti) OR ('stomach cancer':ab,ti) OR ('gastric cancer':ab,ti) OR ('gastric neoplasms':ab,ti) OR ('stomach neoplasm':ab,ti)) AND (('laparoscopy'/exp) OR ('laparoscopic surgery':ab,ti) OR ('laparoscopic gastrectomy':ab,ti) OR ('laparoscopic approach':ab,ti) OR ('minimally invasive procedure'/exp) OR ('minimally invasive surgery':ab,ti))AND ((‘open gastrectomy’/exp) OR (‘open gastrectomy’:ab,ti) OR (‘open surgery’:ab,ti) OR (‘open approach’:ab,ti) OR (‘open procedure’:ab,ti))

Additional file 2-3 Search strategy: Web of Science

(((AB=(gastric cancer OR stomach cancer OR cancer of stomach OR stomach neoplas* OR gastric carcinoma*)) AND AB=(laparoscopic surgery OR laparoscopic gastrectomy OR laparoscopic approach OR laparoscp* gastrectomy OR minimally invasive procedure OR 'minimally invasive surgery)) AND AB=(open gastrectomy OR open gastrectomy OR open surgery OR open approach OR open procedure))

Additional file 2-4 Search strategy: Cochrane Center Register of Controlled Trials

(MeSH descriptor: [Stomach Neoplasms] this term only OR (gastric cancer):ti,ab,kw OR (stomach cancer):ti,ab,kw OR (gastric carcinoma*):ti,ab,kw) AND (MeSH descriptor: [Laparoscopy] this term only OR (laparoscopic gastrectomy):ti,ab,kw OR (laparoscop* surgery):ti,ab,kw OR (minimally invasive gastrectomy):ti,ab,kw OR (minimally invasive surgery):ti,ab,kw OR (minimally invasive procedure):ti,ab,kw) AND ((open gastrectomy):ti,ab,kw OR (open surgery):ti,ab,kw OR (open approach):ti,ab,kw))
